# Supplementary material for: Recruitment of TBK1 to cytosol‐invading Salmonella induces WIPI2‐dependent antibacterial autophagy
Source: EMBO J. 2016 Jul 1;35(16):1779–92. doi: 10.15252/embj.201694491 (PMC5010046; doi:10.15252/embj.201694491)

Recruitment of TBK1 to cytosol-invading Salmonella induces WIPI2-dependent antibacterial autophagy

Teresa L. Thurston[1,2], Keith B. Boyle[1], Mark Allen[3], Ben Ravenhill[1], Maryia Karpiyevich[1], Stuart Bloor[1,4], Annie Kaul[1], Jessica Noad[1], Agnes Foeglein[1], Sophie Matthews[2], David Komander[1], Mark Bycroft[3], Felix Randow[1,5,6]

[1] MRC Laboratory of Molecular Biology, Division of Protein and Nucleic Acid Chemistry, Francis Crick Avenue, Cambridge CB25 9AB, UK

[2] MRC Centre for Molecular Bacteriology and Infection, Imperial College London, Flowers Building, Exhibition Road, London SW7 2AZ, United Kingdom

[3] MRC Laboratory of Molecular Biology, Division of Structural Studies, Francis Crick Avenue, Cambridge CB25 9AB, UK

[4] current address: University of Cambridge, Cambridge Institute for Medical Research, Cambridge, CB2 0XY, UK

[5] University of Cambridge, Department of Medicine, Addenbrooke's Hospital, Cambridge CB2 0QQ, UK

[6] To whom correspondence should be addressed. Tel: +44 1223 267161; Fax: +44 1223 268306; Email: randow@mrc-lmb.cam.ac.uk

Appendix Figures

Figure S1

Figure S2

Figure S3

Appendix Figure S1. Chemical shift mapping of the interaction between the NDP52 zinc finger and ubiquitin.

A) Overlay of the $^{1}H/^{15}N$ HSQC spectrum of 1mM ^{15}N labeled NDP52 zinc finger in the absence (black) or presence (red) of 2mM ubiquitin.

B) Chemical shifts of NDP52 residue upon addition of ubiquitin.

C) Overlay of the $^{1}H/^{15}N$ HSQC spectrum of 1mM ^{15}N ubiquitin in the absence (black) of presence (red) of 2mM NDP52 zinc finger

D) Chemical shift of ubiquitin residues upon addition of NDP52 zinc finger. Chemical shift changes for amide nitrogen (black) and amide proton (white) resonances are given in Hz. Yellow bars residues of ubiquitin whose peaks are broadened upon addition of NDP52.

Appendix Figure S2. Chemical shift changes of residues in the NDP52 zinc finger as a function of ubiquitin concentration

A-B) Overlay of sections of the$^{1}H/^{15}N$ HSQC spectra of ^{15}N labeled NDP52 zinc finger. Peaks correspond to I436 (**A**) and H440 (**B**) at different concentrations of unlabeled ubiquitin.

C) Chemical shifts in dependence of ubiquitin concentration. The dissociation constants were obtained by fitting the data to a single site-binding model.

Appendix Figure S3. $^{1}H/^{15}N$ HSQC spectra of the NDP52 zinc finger.

Wild type (black) and D439K (red).

Appendix Figure S1

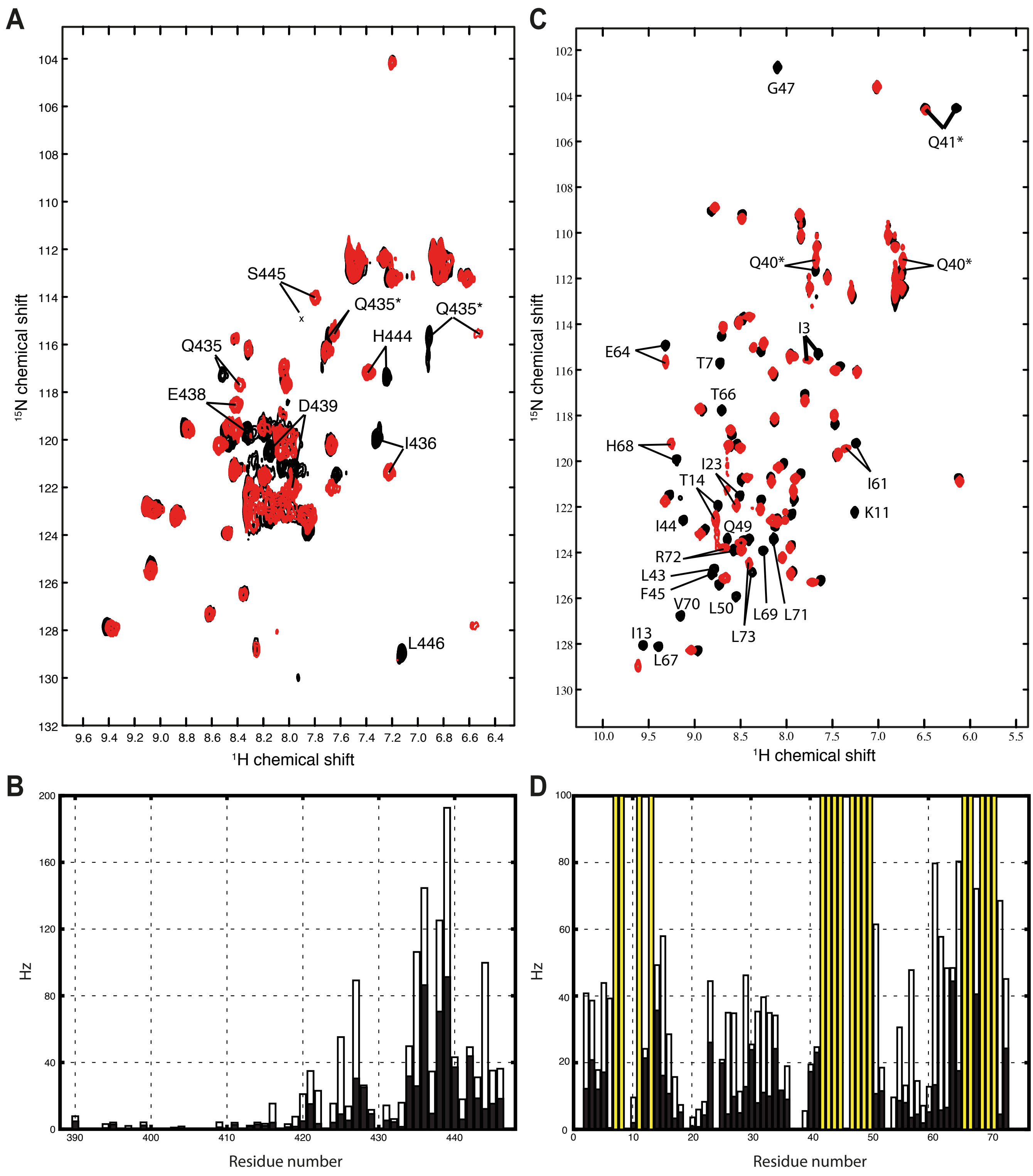

Appendix Figure S2

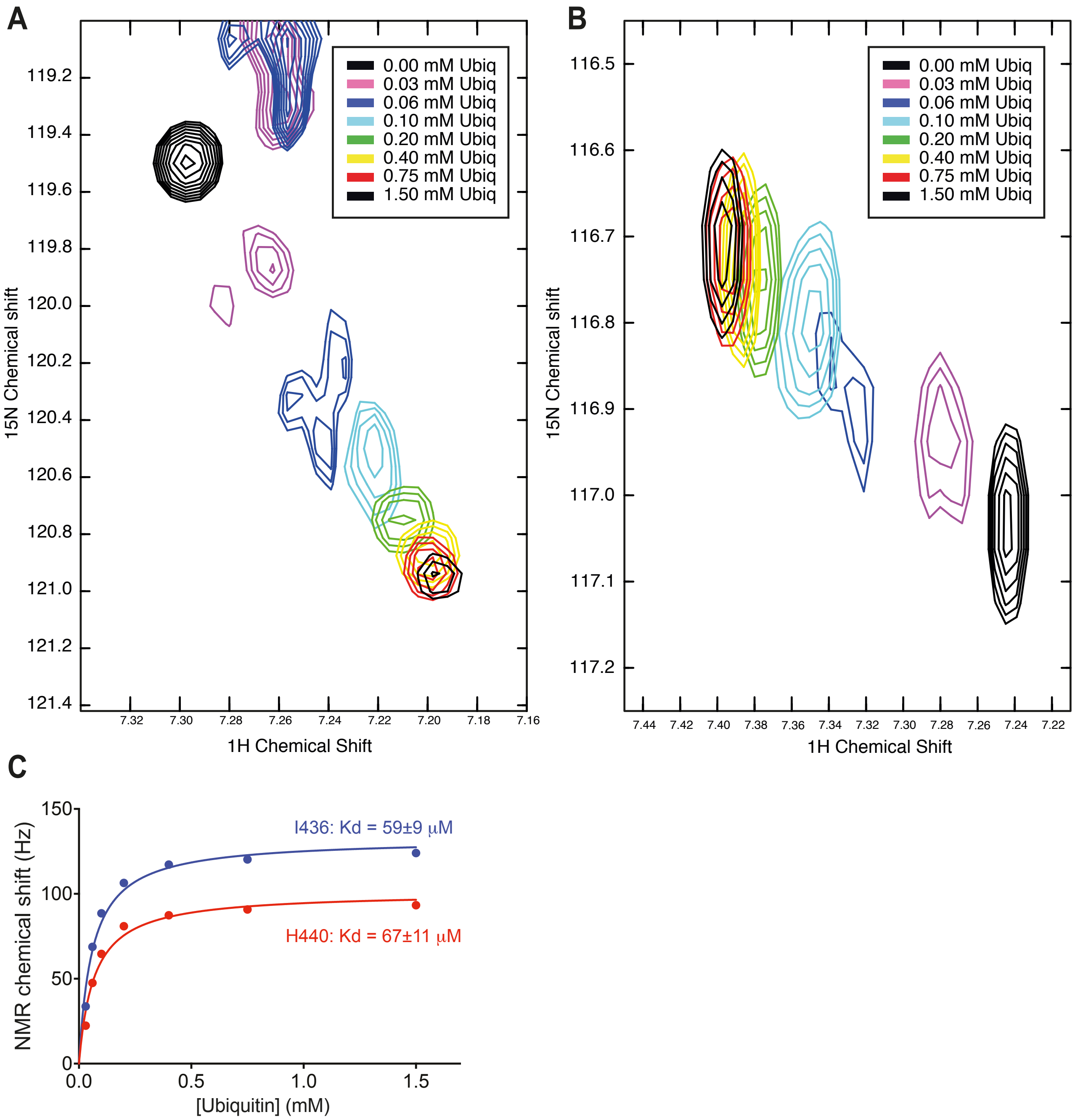

Appendix Figure S3

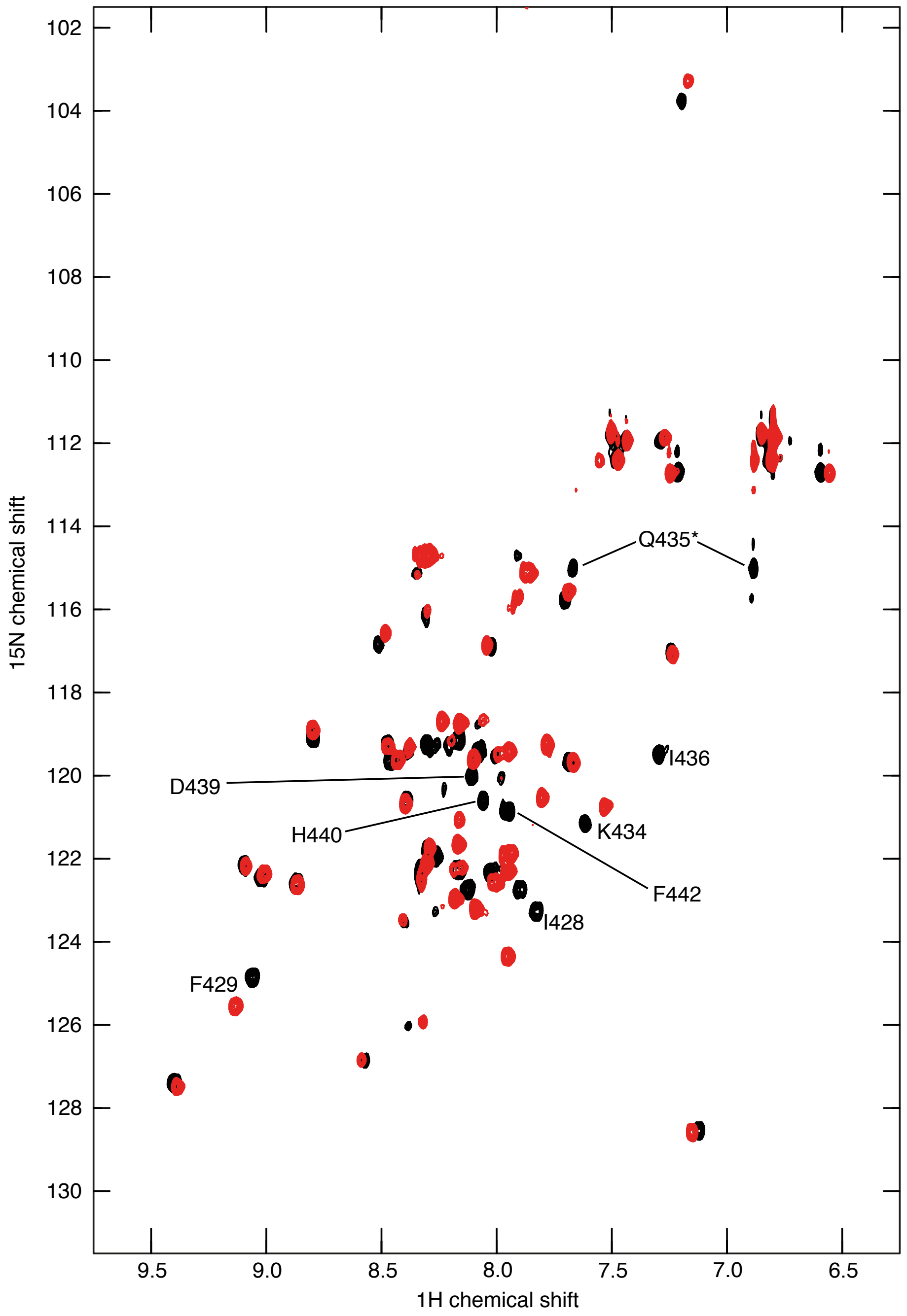

Supplement: Supplementary file 1 — Appendix [file EMBJ-35-1779-s001.pdf]
