# Supplementary material for: Recruitment of TBK1 to cytosol‐invading Salmonella induces WIPI2‐dependent antibacterial autophagy
Source: EMBO J. 2016 Jul 1;35(16):1779–92. doi: 10.15252/embj.201694491 (PMC5010046; doi:10.15252/embj.201694491)
Supplement: Supplementary file 2 — Expanded View Figures PDF [file EMBJ-35-1779-s002.pdf]

Expanded View Figures

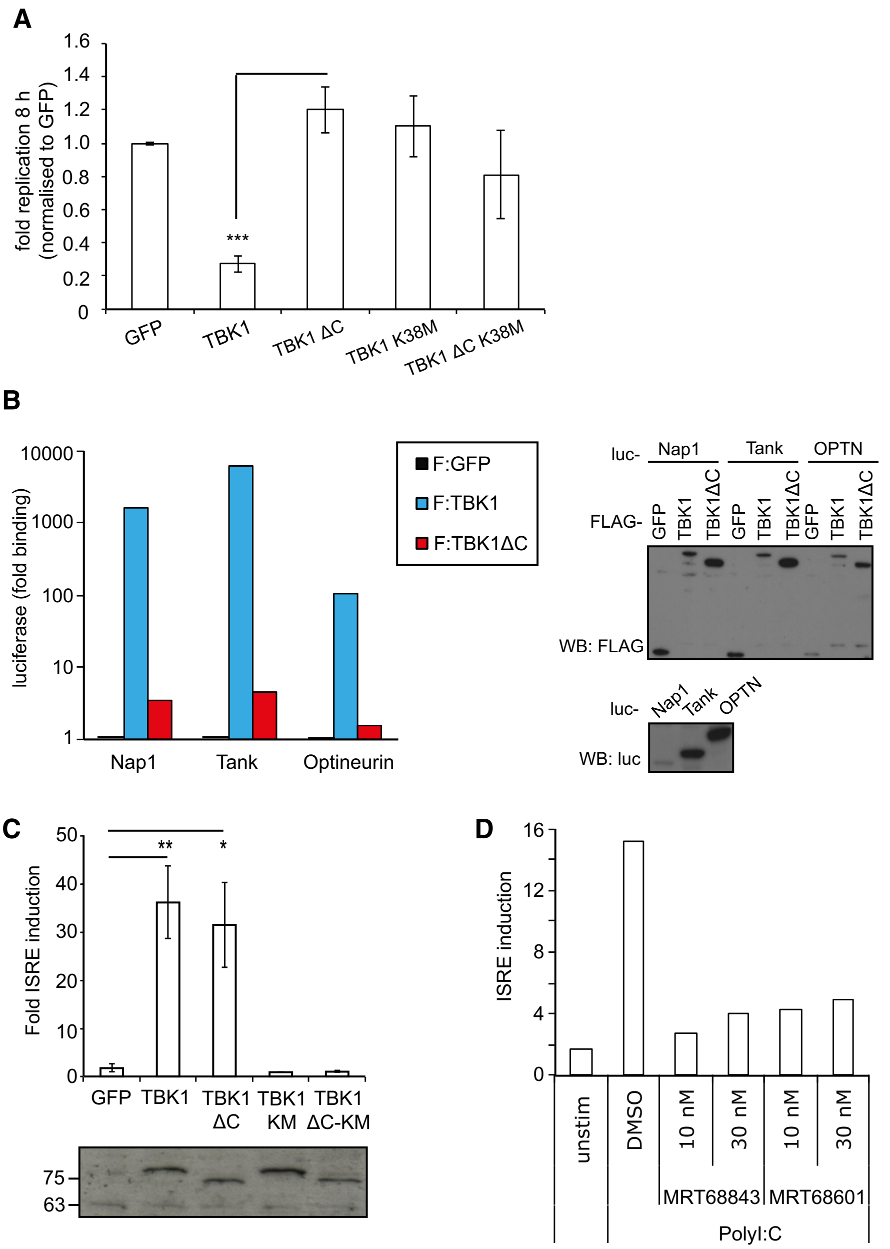

Figure EV1. Characterization of TBK1 alleles.

A Replication of *Salmonella enterica* serovar Typhimurium in *Tbk1*$^{-/-}$ MEFs complemented with the indicated Flag-tagged TBK1$_{\Delta C}$ fusion proteins. Replication normalized to cells expressing GFP. Mean and SEM. $N = 3$, ***$P < 0.001$ one-way ANOVA with Dunnett's multiple comparisons test.

B Lumier binding assay. Binding of luciferase-tagged Nap1, Tank, or optineurin to beads coated with Flag:GFP, Flag:TBK1, or Flag:TBK1$_{\Delta C}$. Western blot for luciferase-tagged or Flag-tagged proteins in total cell lysates.

C ISRE reporter induction upon transient expression of the indicated Flag:TBK1 alleles into 293ET cells. Western blot for Flag-tagged proteins. Mean and SEM of four independent experiments. *$P < 0.05$, **$P < 0.01$, Student's *t*-test.

D ISRE reporter induction upon stimulation of 293ET cells with polyI:C (8 h) in the presence of DMSO or TBK1 inhibitors MRT68843 or MRT68601.

Figure EV2. Recruitment of WIPI1 and WIPI2B to *Salmonella enterica* serovar Typhimurium requires TBK1 but not optineurin.

A Western blots of GFP-tagged WIPI alleles and Flag-tagged TBK1 alleles corresponding to Fig 2B.

B Percentage of WIPI2-positive *S.* Typhimurium at 1 h p.i., in *Tbk1*$^{-/-}$ MEFs complemented with the indicated Flag-tagged TBK1 proteins. Wortmannin (Wort) was added as indicated at 100 nM. Mean and SEM. $N = 3$, *$P < 0.05$, one-way ANOVA with Dunnett's multiple comparisons test. Western blot of TBK1 alleles in post-nuclear lysates.

C, D Analysis of MEFs, stably expressing GFP:WIPI1 or GFP:WIPI2B, treated with the indicated siRNAs and infected with *S.* Typhimurium for 1 h. Mean and SEM. $N = 3$. Western blot for optineurin and tubulin upon the indicated siRNA treatment.

▶

© 2016 MRC Laboratory of Molecular Biology

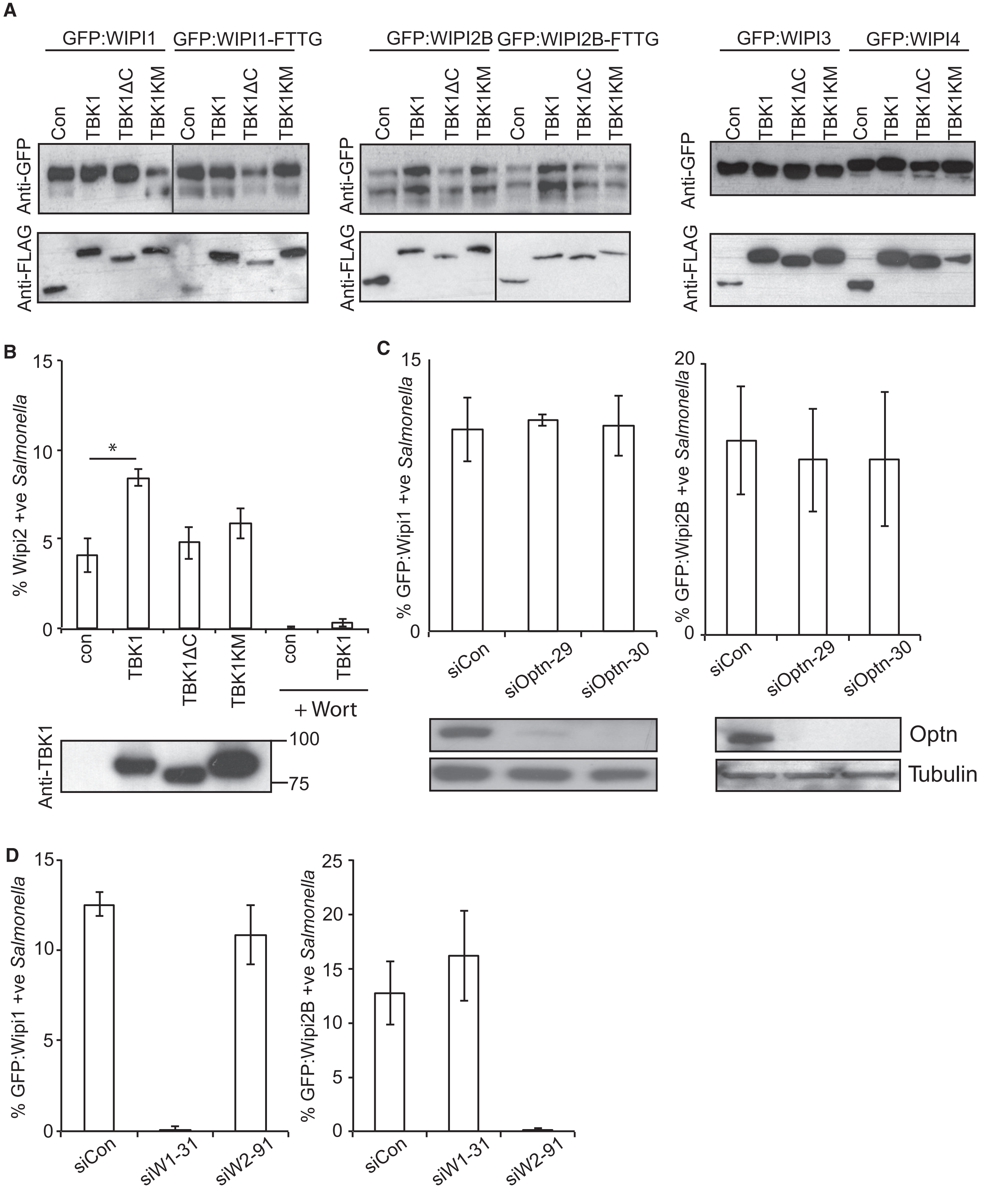

Figure EV2.

 © 2016 MRC Laboratory of Molecular Biology

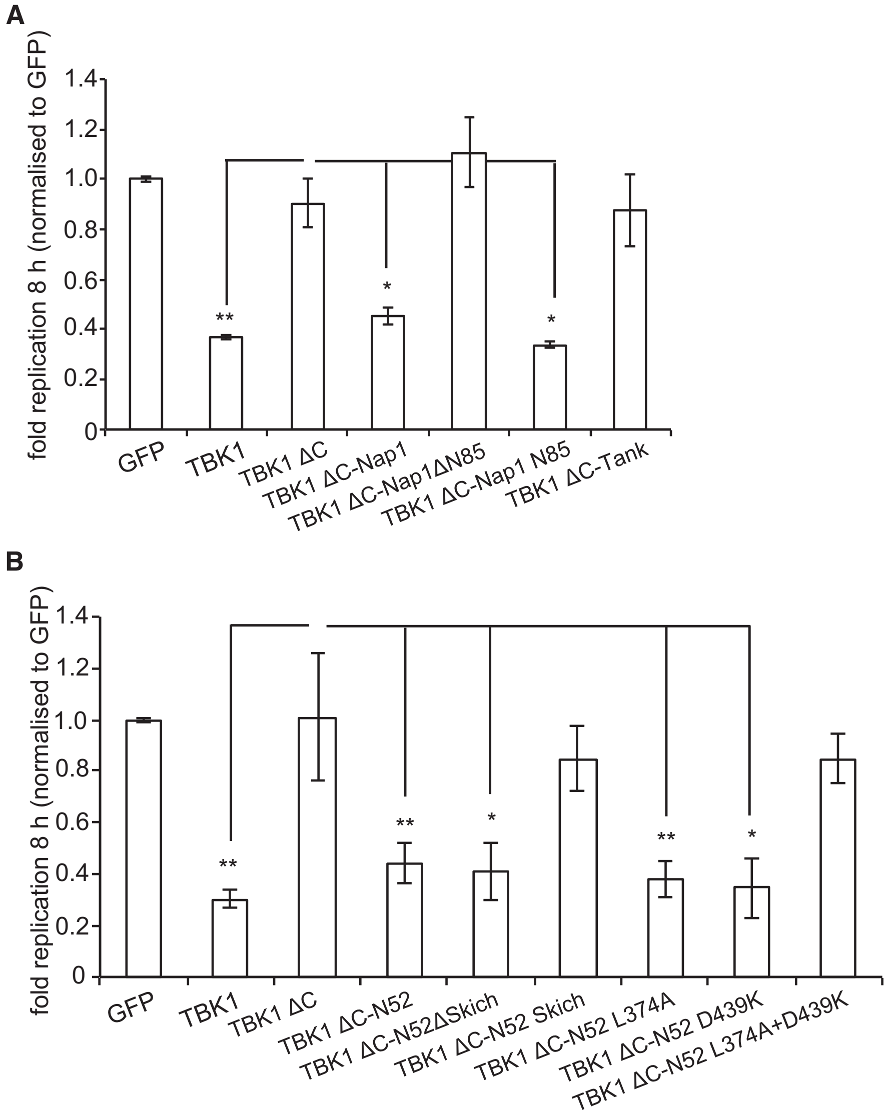

Figure EV3. Recruitment of TBK1 to *Salmonella enterica* serovar Typhimurium via NAP1 or NDP52, but not TANK, restricts bacterial proliferation.

A, B Replication of *S.* Typhimurium in *Tbk1*$^{-/-}$ MEFs complemented with the indicated Flag-tagged TBK1$_{\Delta C}$ alleles. Replication normalized to cells expressing GFP. Mean and SEM. $N = 3$, *$P < 0.05$, **$P < 0.01$, one-way ANOVA with Dunnett's multiple comparisons test.

© 2016 MRC Laboratory of Molecular Biology

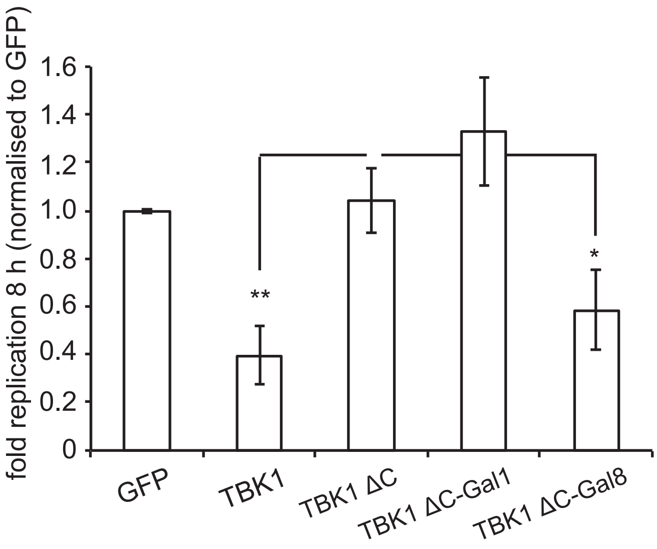

Figure EV4. Recruitment of TBK1 to *Salmonella enterica* serovar Typhimurium via galectin restricts bacterial proliferation.
Replication of *S.* Typhimurium in *Tbk1*$^{-/-}$ MEFs complemented with the indicated Flag-tagged TBK1$_{\Delta C}$ fusion proteins. Replication normalized to cells expressing GFP. Mean and SEM. $N = 4$, *$P < 0.05$, **$P < 0.01$, one-way ANOVA with Dunnett's multiple comparisons test.

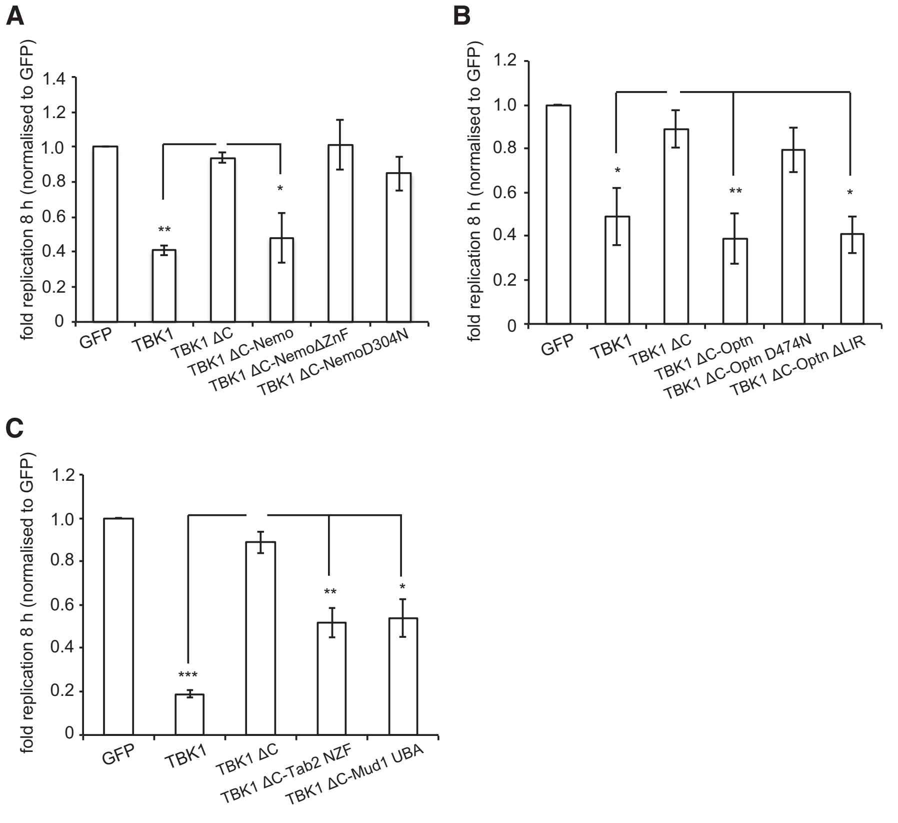

Figure EV5. Recruitment of TBK1 to *Salmonella enterica* serovar Typhimurium via ubiquitin restricts bacterial proliferation.

A–C Replication of *S.* Typhimurium in *Tbk1*$^{-/-}$ MEFs complemented with the indicated Flag-tagged TBK1$_{\Delta C}$ fusion proteins. Replication normalized to cells expressing GFP. Mean and SEM. $N = 4$, *$P < 0.05$, **$P < 0.01$, ***$P < 0.001$, one-way ANOVA with Dunnett's multiple comparisons test.

© 2016 MRC Laboratory of Molecular Biology
